# Physiological, Genomic and Transcriptomic Analyses Reveal the Adaptation Mechanisms of *Acidiella bohemica* to Extreme Acid Mine Drainage Environments

**DOI:** 10.3389/fmicb.2021.705839

**Published:** 2021-07-08

**Authors:** Shu-ning Ou, Jie-Liang Liang, Xiao-min Jiang, Bin Liao, Pu Jia, Wen-sheng Shu, Jin-tian Li

**Affiliations:** ^1^Institute of Ecological Science, Guangzhou Key Laboratory of Subtropical Biodiversity and Biomonitoring, Guangdong Provincial Key Laboratory of Biotechnology for Plant Development, School of Life Sciences, South China Normal University, Guangzhou, China; ^2^School of Life Sciences, Sun Yat-sen University, Guangzhou, China

**Keywords:** *Acidiella bohemica*, fungi, acid mine drainage, physiology, genome, transcriptome

## Abstract

Fungi in acid mine drainage (AMD) environments are of great concern due to their potentials of decomposing organic carbon, absorbing heavy metals and reducing AMD acidity. Based on morphological analysis and ITS/18S high-throughput sequencing technology, previous studies have provided deep insights into the diversity and community composition of fungi in AMD environments. However, knowledge about physiology, metabolic potential and transcriptome profiles of fungi inhabiting AMD environments is still scarce. Here, we reported the physiological, genomic, and transcriptomic characterization of *Acidiella bohemica* SYSU C17045 to improve our understanding of the physiological, genomic, and transcriptomic mechanisms underlying fungal adaptation to AMD environments. *A. bohemica* was isolated from an AMD environment, which has been proved to be an acidophilic fungus in this study. The surface of *A. bohemica* cultured in AMD solutions was covered with a large number of minerals such as jarosite. We thus inferred that the *A. bohemica* might have the potential of biologically induced mineralization. Taking advantage of PacBio single-molecule real-time sequencing, we obtained the high-quality genome sequences of *A. bohemica* (50 Mbp). To our knowledge, this was the first attempt to employ a third-generation sequencing technology to explore the genomic traits of fungi isolated from AMD environments. Moreover, our transcriptomic analysis revealed that a series of genes in the *A. bohemica* genome were related to its metabolic pathways of C, N, S, and Fe as well as its adaptation mechanisms, including the response to acid stress and the resistance to heavy metals. Overall, our physiological, genomic, and transcriptomic data provide a foundation for understanding the metabolic potential and adaptation mechanisms of fungi in AMD environments.

## Introduction

Acid mine drainage (AMD) ecosystems include distinct environments with AMD solutions, sediments and biofilms. The AMD environments is mainly formed by oxidation of pyrite and other metal sulfides exposed to air and oxygenated water. The spontaneous formation of this process is very slow. However, the microorganisms with oxidation functions of iron and sulfur in AMD environments can greatly accelerate the oxidation processes of metal sulfides, the release of heavy metals and the production of sulfuric acid to increase rates of AMD formation (Nordstrom and Alpers, 1999; Barrie and Hallberg, 2009; Denef et al., 2010; Méndez-García et al., 2014). The AMD environments are characteristic of extremely low pH, high heavy metal and sulfate concentrations. If they were not treated, the AMD solutions will have a critical impact on our habitats after entering rivers, lakes or soils (Johnson and Hallberg, 2005; Larson, 2014). In such an extreme AMD environment, microbial communities have low diversity and simple relationships between organisms (Rothschild and Mancinelli, 2001; Denef and Banfield, 2012). Even so, during a long-term evolution, some beneficial microorganisms own special mechanisms that can cope with various extremely environmental stress such as low pH and high heavy metal concentrations. Such a complete and simple microbial system is regarded as a model system for studying microbial ecology and evolution (Denef et al., 2010). Studying microorganisms from AMD environments is conducive to develop the bioremediation and treatment strategies for AMD pollution (Chen et al., 2016).

In the past few decades, studies have shown that there are different functional groups of microorganisms in AMD environments, including bacteria, archaea, and fungi. Isolation and cultivation methods, SSU rRNA/ITS molecular marker gene sequencing and meta-omics analyses, have dramatically improved our knowledge of microbial taxa and diversity in AMD environments (Baker and Banfield, 2003; Denef et al., 2010; Chen et al., 2016; Huang et al., 2016). However, there are numerous studies on bacteria and archaea, few on fungi. As early as the mid-1970s, an acidophilic fungus named *Scytalidium acidophilum* (now *Acidomyces acidophilus*) was successfully isolated from AMD environments (Sigler and Carmichael, 1974). 18S rRNA and β-tubulin gene cloning and sequencing were used to analyze the diversity of eukaryotic microbes in AMD environments of the Richmond mine and isolated a novel fungus *Acidomyces richmondensis* belonging to the *Dothideomycetes*. This was the first combination of sequencing and fluorescence *in situ* hybridization (FISH) to prove the existence of specific microbial groups (Baker et al., 2004). Hujslová isolated *Acidiella bohemica* (*Dothideomycetes*, *Capnodiales*, *Teratosphaeriaceae*) (Hujslová et al., 2013) and *Acidothrix acidophila*, *Soosiella minima*, and *Acidea extrema* (Hujslová et al., 2014) from extremely acidic soil in the Czechia. Recently, two *Penicillium* isolates, strains ShG4B and ShG4C were isolated from extremely metal-rich waters at a mine in Russia (Glukhova et al., 2018). To date, more than 20 species of fungi have been isolated and cultured from AMD and other acid environments (Supplementary Table 1) (Starkey and Waksman, 1943; Sinclair and Herring, 1975; Cánovas et al., 2003; Baker et al., 2004; Hölker et al., 2004; Selbmann et al., 2008; Luo et al., 2009; Yamazaki et al., 2010; Hujslová et al., 2013, 2014; Glukhova et al., 2018). However, most of the fungi are identified with traditional morphological analysis methods. Studies on fungi of AMD environments are mainly limited to the morphological description and community composition of fungal species in pure culture. We rarely obtained the high-quality genomes of fungi isolated from AMD environments and the ecological role of fungi in molecular level is still unknown.

In extremely acid environments rich in heavy metals, the survival and reproduction of fungi require metabolic potential and a series of heavy metal and acid adaptation mechanisms (Gadd, 2007; Gadd and Raven, 2010; Oggerin et al., 2013; Mosier et al., 2016). As a major contributor in AMD environments (Gunde-Cimerman et al., 2009), fungi can adapt to low pH (pHs 0.8–1.38) and high heavy metal concentrations (up to 269 mM Fe^2+^, 16.8 mM Zn, 8.5 mM As, and 4.1 mM Cu), and can decompose organic carbon and directly participate in ferric or sulfate reduction processes (Ottow and Von Klopotek, 1969; Baker et al., 2004). They are also able to absorb heavy metal and organic pollutants to repair the AMD environments (Nakatsu and Hutchinson, 1988; Shiomi et al., 2004; Das et al., 2009). It is considered that covering the surface of AMD solutions with fungal hyphae is a good way to remove heavy metals (Shiomi et al., 2004). In addition, researchers obtained the *A. richmondensis* genome by the second-generation sequencing technology and revealed the carbon and nitrogen cycles as well as adaptation mechanisms (Mosier et al., 2016). Although progress has been made, there are still more fungi isolated from AMD environments, whose genomes, metabolic pathways, and adaptation mechanisms have not been understood. In the past, fungal genomes were generally sequenced by second-generation sequencing technology, but rarely by single-molecule real-time sequencing. In fact, single-molecular real-time sequencing can produce much longer reads than the second-generation sequencing. It can recognize the regions rich in repeat fragments in the genome, which is helpful for the assembly of *de novo* genome (Fraser et al., 2002; Faino et al., 2015).

Here, we used AMD solutions to culture the *A. bohemica* SYSU C17045 isolated from sediments that collected from Fankou Pb-Zn mine. *A. bohemica* was first isolated and characterized by Hujslová et al. (2013). In this study, we reported that the *A. bohemica* was sequenced and assembled by PacBio single-molecule real-time sequencing. Based on physiological, genomic, and transcriptomic analyses, we revealed several metabolic pathways of carbon, nitrogen, sulfur, and iron as well as potential environmental adaptation mechanisms of *A. bohemica*. In addition, we compared the similarities and differences between the *A. bohemica* genome and genomes of its closely related species. This study will enrich the database of fungal genomes and explain the metabolic potentials of *A. bohemica* with biologically induced mineralization potential and its extremely environmental adaptation mechanisms.

## Materials and Methods

### Sample Collection, Microbial Culture, and Strain Characterization

This study was conducted in the tailings impoundment of the Fankou Pb-Zn mine, Renhua, Guangdong Province (N25°2′57.5″, E113°39′34.1″’), China. This region is a subtropical monsoon climate zone, with annual precipitation around 1,670 mm and annual mean temperature of 18°C (Huang et al., 2011). Sediment samples were collected with sterile 50 ml centrifuge tubes on April 20, 2016 and kept under refrigeration until arrival at laboratory where they were stored at 4°C before processing. AMD samples were collected with sterile serum bottles and immediately kept on ice for transport to the laboratory. After treated with phosphate buffer, sediment samples (pH 2.2) were coated on optimized 9K solid media (pH 3) for cultivation. Subsequently, the strain was isolated and purified for PCR amplification and full-length ITS sequencing, after cultured at 25°C for one month (Kuang et al., 2012). The obtained sequences were compared to the reference sequences of *A. bohemica*, *Fodinomyces uranophilus*, and *Penidiella* sp. (JQ172757.2, KU365882.1, and AB845352.1) as found on NCBI by using BLAST to determine the taxonomic status of this species (Altschul et al., 1997).

### Morphological and Physiological Analyses

We used potato-dextrose agar (PDA) to incubate the *A. bohemica* and used 10 pH gradients (1–10) to identify the pH adaptability range of this strain. With 1% inoculation volume, the strain was incubated at 25°C for 7 days. We observed the growth status of this strain and determined the dry weight after centrifugation and air drying (Hujslová et al., 2013). Furthermore, AMD samples were vertically filtered through 0.8 μm and 0.22 μm polyethersulfone membrane filters, and then filtered through tangential flow with 1,000 and 30 kDa membranes to obtain the filtrate. The filtrate was added with 3% glucose to cultivate this strain for 30 days and set 3 blank control groups without inoculating this strain (Kuang et al., 2012). After removing impurities, the precipitates were performed into SEM samples to observe using the thermal field emission scanning electron microscope (Quanta 400, FEI, Netherlands) (Nguyen and Harbison, 2017), and to analyze using the X-ray spectrometer (EDS, INCA, Oxford, United Kingdom). Moreover, we used transmission electron microscopy (JEM-100CX-II, JEOL, Japan) to observe ultrathin sectioning. The precipitates were washed with sterile water to remove the culture solutions and other impurities. Then the precipitates were washed with 5% SDS and treated with ultrasonic wave, centrifuged to remove most of the cell debris. After freeze-drying and through a 300 mesh sieve, the precipitates were analyzed by X-ray powder diffractometer (Empyrean, Netherlands) (Tamura and Gilbert, 2013). Then, the X-ray diffraction pattern was analyzed using Jade 5.0 software (Li et al., 2012). After microwave digestion, metal and S elements in culture solutions were determined by the inductively coupled plasma emission spectrometer (ICP-OES, Optima 2100DV, Perkin-Elmer, United States). The concentrations of ferric ions (Fe^3+^) and ferrous ions (Fe^2+^) were determined by 1, 10-phenanthroline colorimetric method (Hill et al., 1978).

### DNA Extraction, Sequencing, and Genome Analysis

We collected this strain for DNA extraction and sequencing to obtain genomic data when the growth of the strain reached in logarithmic phase. Culture solutions were centrifuged to collect this strain and then washed with sterile water for three times. The strain was manually homogenized with 500 μl 65°C pre-warmed cetyltrimethyl ammonium bromide (CTAB) buffer. Homogenized samples were incubated at 65°C for 30 min, further homogenized, and cooled on ice for 5 min. The homogenate was added with RNAase and incubated at 37°C for 15 min. An equal volume of phenol: chloroform: isoamyl alcohol (25:24:1) was added to the centrifuge tube. Samples were centrifuged for 10 min at 12,000 × *g* at 4°C after mixing. The aqueous supernatant was removed and an equal volume of chloroform: isoamyl alcohol (24:1) was added to the tube, mixed, and centrifuged as before. The supernatant was removed again and DNA was precipitated with 0.6X volume of isopropanol and 0.1X volume 3M CHCOONa at −20°C for 20 min, mixed, and centrifuged as before. The supernatant was removed and the pellet was washed with 70% ethanol, centrifuged for 10 min, dried. DNA was dissolved with ddH_2_O, mixed, and stored at −20°C (Mosier et al., 2016).

Genomic DNA was randomly broken into 500 and 800 bp by Covaris M220. We utilized the NEBNext^®^ Ultra^TM^ II DNA Library Prep Kit for Illumina^®^ (NEW ENGLAND BioLabs, # E7645) to construct the DNA libraries. We performed paired-end 250 bp sequencing using the Illumina Miseq sequencer and obtained a total of 9.17 Gb data. We used the SMRTbell^TM^ Template Prep Kit (Pacific Bioscience, #100-259-100) to construct a 20 kb SMRT long library, and then used PacBio sequel platform to sequence. Based on PacBio sequel sequencing data, Falcon and Falcon-unzip (version 0.1.3) (Chin et al., 2016) were used for genome assembly. In addition, we utilized the existing highly conserved and low-copy genes to evaluate genome integrity. CEGMA version 2.5 was applied to evaluate the genomic integrity according to 248 eukaryotic core genes (Parra et al., 2007). Moreover, BUSCO (Benchmarking Universal Single-Copy Orthologs) was used for quality assessment (Simão et al., 2015). RepeatModeler was utilized to establish the transposon model to predict the repeat sequences (Saha et al., 2008). Published protein and genome sequences of 12 species were downloaded from NCBI and JGI (Supplementary Table 2). In our data set, there were species of different families (Teratosphaeriaceae and Mycosphaerellaceae affiliated with Capnodiales), orders (Capnodiales and Dothideales) and classes (Dothideomycetes), and *Aspergillus niger* was the outgroup. According to the feature frequency profile (FFP) method, the frequencies of 13 l-mer features were selected to construct the concept tree using the consensus toolkit in PHYLIP package. Jackknife test was used to evaluate the sampling deviation of main groups and verify the reliability of tree structure (Choi and Kim, 2017). OrthoMCL version 2.0.9 (Li et al., 2003) was used to analyze the homologous genes of four species, *A. bohemica*, *Baudoinia panamericana*, *Dothistoma septosporum*, and *A. richmondensis*. Based on sequence similarity, the OrthoMCL could classify four proteomes into ortholog groups, in-parallel groups and co-orthologs.

### RNA Extraction, Sequencing, and Transcriptome Analysis

We also collected this strain for RNA extraction and sequencing to obtain transcriptomic data, when the growth of this strain reached in logarithmic phase. Total RNA was isolated by using a RNeasy plus mini kit (cat. no. 74903) according to the manufacturer’s protocol. After poly-A selection to enrich mRNA and reverse transcription to construct cDNA libraries, the Hiseq X Ten platform performed double-ended 150 bp sequencing to obtain transcriptomic data. The RNA library construction and sequencing were completed by Aiji Biotechnology Co., Ltd. (Guangzhou). For the prediction of RNA-seq data, Trinity software (version 2.8.4) was used to splice the sequence reads of transcriptome (Grabherr et al., 2011; Haas et al., 2013), and 37,996 contigs were assembled from scratch. In order to splice the transcriptomic data based on a reference genome, Tophat2 was applied to align the reads of RNA-seq to the *A. bohemica* genome assembly (Kim et al., 2013). Then the alignment results were assembled by using Cufflinks (Roberts et al., 2011). GeneMark-ET and Augustus of BRAKER software were utilized for *de novo* predictions of *A. Bohemica* genes, which included PASA (Program to Assemble Spliced Alignments) software regarding transcriptomic data as a training set to improve the accuracy of prediction (Haas, 2003). Finally, EVidenceModeler (EVM) was applied to integrate the above prediction results, and PASA was used to optimize the integration results (Haas et al., 2008, 2011). Combined with genomic and transcriptomic data, the expression of predicted genes was calculated by using RSEM software (Li and Dewey, 2011). Predicted genes were aligned to NCBI-nr, KOG, KEGG, and Pfam databases. The prediction and classification data of carbohydrate-active enzymes (CAZymes) came from CAZy database^1^, and the annotation of CAZymes was based on HMM model using dbCAN. We added eight species belonging to *Hysteriales* and *Pleosporales* according to different lifestyles to compare with the original species, and the R studio was used to cluster the CAZyme family and species, using the Euclidean distance matrix.

## Results

### Morphological and Physiological Characteristics

**FIGURE 1:**
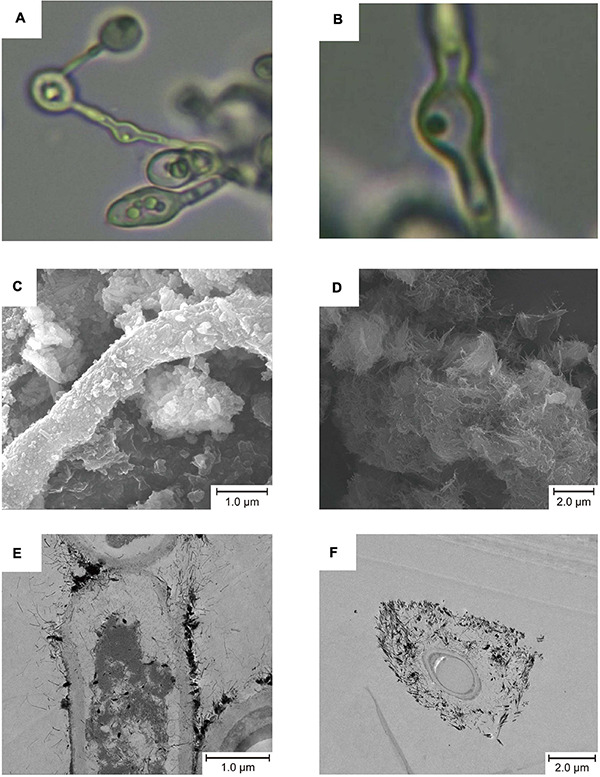
Micro morphology of *A. bohemica* **(A,B)**, SEM micrographs **(C,D)**, and TEM micrographs **(E,F)**. **(A,B)** The mycelial morphology of *A. bohemica*. **(C)** Particles deposited on the fungal hyphae. **(D)** Acicular precipitates gathered. **(E)** Mineralization started on the surface of the fungal cell wall. **(F)** The fungal cell was completely surrounded by minerals.

We obtained 31 pure culture of fungi from sediment samples collected from Fankou Pb-Zn mine tailing, 20 of which belong to *A. bohemica* SYSU C17045. Taxonomically, it belongs to the family Teratosphaeriaceae (*Capnodiales*, *Dothideomycetes*, and *Ascomycota*). The colonies of *A. bohemica*, a filamentous fungus, slowly grew and centrally heaped. In acidic 9 k medium, the mycelia were composed of separated, colorless or pale brown hyphae. The conidia were formed by fragmentation and they were oblong or swollen with truncated ends (Figures 1A,B). The result of pH adaptability experiment showed that *A. bohemica* SYSU C17045 could grow in the range of pH 2–8, among which pH 3 was the most suitable for growing of this strain, but pH 1, pH 9–10 were inhibited (Supplementary Figure 1).

Since *A. bohemica* was inoculated with AMD solutions and glucose liquid, it grew and propagated in a good trend in early stage, and the surface of colonies appeared dark brown and black. In addition to the clumped colonies in the liquid medium, a part of colonies grew on the wall of the erlenmeyer flask. Ten days later, yellow substances could be seen on the surface of colonies. After that, no obvious growth and reproduction of colonies were observed. Twenty-five days later, a large amount of yellow substances could be observed on the surface of colonies. Nevertheless, there were still some brown-black colonies that were exposed and unwrapped. After 50 days of cultivation, the colonies were clearly wrapped with yellow substances. Taken out a part of yellow substances and poked with a bamboo stick, they were very tightly bound with this strain, and the yellow substances did not dissolve under acidic conditions. It could be seen from the results of SEM that some fungal hyphae had some particles on the surface (Figure 1C) and some were attached with a large number of acicular precipitates (Figure 1D). In TEM micrographs, mineralization started on the surface of the fungal cell walls, and then cells were completely surrounded by minerals (Figures 1E,F). The physiological analysis of the culture solutions showed that compared with the non-inoculated control, the Fe elements in the culture solutions were greatly reduced by 20.7%, and the S and Ca elements were also significantly reduced (Supplementary Table 3).

### Express Genes of Environmental Adaptation in *A. bohemica*

**FIGURE 2:**
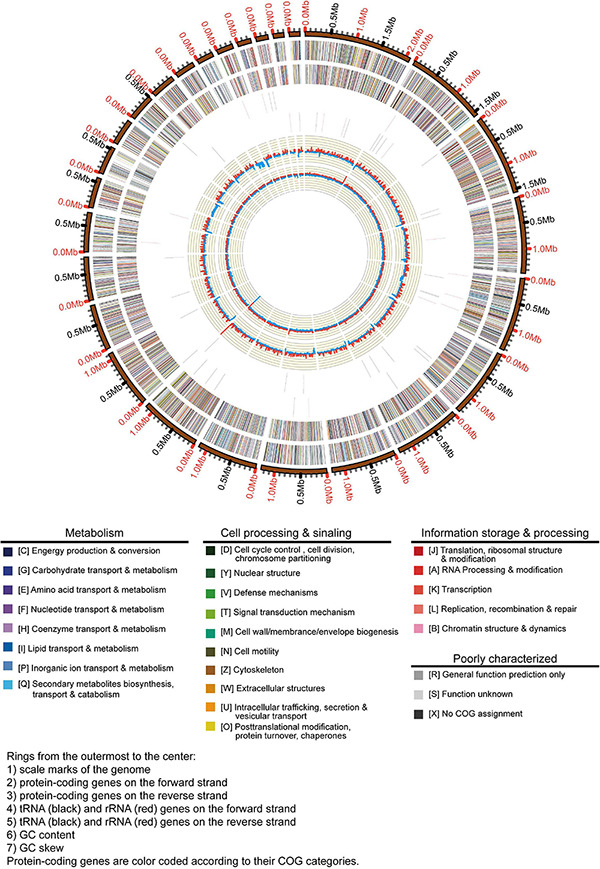
Graphical map of the *A. bohemica* genome.

In this study, we obtained the whole genome data of *A. bohemica* (Figure 2). The primary contigs included 62 contigs. Among them, the total length of primary contigs was 26.8 Mbp, of which N50 was 1.16 Mbp and the longest primary contigs was 2.1 Mbp. Besides, the GC content of primary contigs was about 56.9%. The *A. bohemica* genome included 10,985 predicted genes (Supplementary Table 4), compared to the *A. richmondensis* genome of 26.8 Mbp with 10,352 genes (Mosier et al., 2016). The quality of genome assembly of *A. bohemica* is better than that of *A. richmondensis*. We performed two different methods to assess the completeness of the *A. bohemica* genome. One was that the existing 248 eukaryotic orthologous genes could be divided these genes into four groups according to their conservativeness. More than 93.0% genes in primary contigs could be completely aligned to these four groups of gene sets. The assessment of the full genome reached 97.6% completeness. Another was based on a database analysis of ascomycete single-copy orthologous genes. The database contains 1,315 ubiquitous single-copy genes. A total of 1,260 complete genes were found in primary contigs. The assessment of the full genome reached 97.8% completeness. These results have confirmed a high completeness of our genome assembly. In *de novo* prediction, GeneMark software predicted a total of 10,001 genes, and Augustus software predicted a total of 10,127 genes. A total of 86.2% of the transcriptomic data were aligned to the whole genome and 79.1% of the sequences could be aligned to the primary contigs using Tophat2. A total of 5,912 transcripts were assembled by Cufflinks. Besides, Trinity and PASA predicted a total of 10,846 genes. After integration and optimization by EVM and PASA respectively, the number of predicted genes in primary contigs was 10,985, and predicted genes in haplotigs were 9,212. Gene annotation and metabolic analysis were mainly aimed at primary contigs. A total of 90.7% of the predicted genes were homologous with reference genes in the NCBI-nr database; 82.2% had corresponding KOG annotation information in the KOG database, and 35.7% could be assigned to the corresponding KEGG pathways in the KEGG database.

#### Carbon, Nitrogen, and Sulfur Cycling

Enzymes involved in their decomposition show significantly functional diversity, due to the huge structural and functional diversity of complex carbohydrates (Supplementary Table 5). The genome harbored a total of 520 CAZymes comprising 100 glycosyltransferases (GTs), 239 glycoside hydrolases (GHs), 100 carbohydrate esterases (CEs), 2 polysaccharide lyases (PLs), 40 Auxiliary Activities (AAs), and 39 carbohydrate-binding modules (CBMs), as well as 45 cellulase genes (families GH1, GH3, GH5, GH6, GH7, GH12, GH30, and GH45). Furthermore, a great many of genes in the *A. bohemica* genome are required for the metabolism of fructose, mannose, galactose, starch, and sucrose. According to the KEGG annotation results, GHs mainly contain alpha-glucosidase (GH31), beta-glucosidase (GH1 and GH39), glucoamylase (GH15), alpha-amylase (GH57), endoglucanase, and glycogen debranching enzyme. The genome also contains genes encoding glycerol-3-phosphate dehydrogenase and glycerol kinase. In addition, the genome encoded Embden-Meyerhof (EMP) pathway, pentose phosphate pathway and tricarboxylic acid cycle (TCA). We also found many enzymes related to respiratory chain in the genome (Supplementary Table 5). Thus, *A. bohemica* may decompose a variety of macromolecular carbon to obtain carbon source in oligotrophic AMD environments.

Likewise, we found the nitrate reductase gene (NR) and nitrite reductase gene (NIT-6) involved in the nitrate assimilation in *A. bohemica* genome (Supplementary Table 5). Besides, glutamate synthase gene (GLT1) and glutamine syntheta gene (*glnA*) were also predicted in *A. bohemica* genome, but no genes related to nitrogen fixation were identified. The *A. bohemica* genome also encoded the cytochrome P450 nitric oxide reductase (P450nor), but no nitrite reductase (NirK, which catalyzes the formation of nitrite to NO) in denitrification pathway was found. Transcriptomic data showed that all above the predicted genes had transcriptional activity.

The related genes encoding the enzymes involved in assimilation sulfate reduction were found in *A. bohemica* (Supplementary Table 5), comprising sulfate adenylyltransferase (sat), adenylylsulfate kinase (cysC), phosphoadenosine phosphosulfate reductase (cysH), sulfate reductase (NADPH) flavoprotein alpha-component (cysJ), sulfite reductase (NADPH) hemoprotein beta-component (cysI), cysteine synthase A (cysK), etc. In addition, transcriptional results provided evidence that these genes might play an important role in sulfur metabolism due to their high expression level.

#### Iron Transformation

The *A. bohemica* genome possessed a wide range of metal transporters aiming at iron (Supplementary Table 5). Iron in the reduced state could be absorbed through a high- or low-affinity system on the plasma membrane of *A. bohemica*. In *A. bohemica*, the high-affinity system consisted of iron transport multicopper oxidase (Fet3) and high-affinity iron transporter (Fth1). Their transcriptional activities were high, with FPKM values of 571 and 775, respectively. Ferrous iron was the only known substrate of this complex. *A. bohemica* also encoded copper chaperone (Atx1) and Cu^2+^-exporting ATPase (Ccc2) to supply oxidation activity for Fet3 by delivering free copper ions. The high-affinity system was composed of low-affinity ferrous iron transport protein (Fet4) and metal iron transporter (Smf). Smf1 is mainly a manganese transporter on the membrane, but it can also transport iron and cobalt. Fet4 is not specific, which can transport not only iron, but also zinc, copper, and calcium. In addition, many iron transporters were also found in vacuoles, including vacuolar Fe^2+^/Mn^2+^ transporter (Ccc1), ferric reductase (Fre6), iron transport multicopper oxidase (Fet5), high-affinity iron transporter (Fth1), and metal iron transporter (Smf3). Fet5, Fth1 and the high-affinity ferrous transport complex (Fet3 and Ftr1) on the plasma membrane are paralogous proteins. These proteins or enzymes in vacuoles did not specifically transport iron, but also zinc, copper, and manganese. There were many iron transporters in mitochondria, containing mitochondrial iron transporter (Mrs4), frataxin (Yfh1), glutaredoxin 3 (Grx3), monothiol glutaredoxin (Grx5), and iron-regulated transcriptional activator (Aft2).

#### Response to Acid Stress

Under the stress of low pH, the *A. bohemica* genome encoded KUP system potassium uptake protein, cation transporters, H^+^ transporting ATPases, Na^+^/H^+^ exchanger, and several symporters. For example, general alpha glucoside is a H^+^ and urea-proton symporter that can prevent the inflow of protons and pump excess protons out of the cells. Transcriptomic data indicated that H^+^ transporting ATPases may be the most active (FPKM value is 1,243). Moreover, the genome also encoded the low-affinity H^+^/Pi symporters (PHO87) and the high-affinity H^+^/Pi symporters (PHO84). In addition, the genome encoded the high-affinity Na^+^/Pi symporter (PHO89) and the low-affinity phosphate transporter (PHO91) on the vacuole membrane, which is essential for the storage and usage of polyphosphates. Propionyl-CoA and acetyl-CoA synthetases encoded in the genome were helpful for the degradation of organic acids. The *A. bohemica* genome contained several genes related to DNA and protein repair. Many genes encoded chaperones, including dnaK, dnaJ, ASF1, hscB, GRPE, PSMG3, and HSP90A. Among them, dnaK and HSP90A have the highest transcriptional activity. The existence of these genes may be related to the maintenance of intracellular pH balance (Supplementary Table 5).

#### Heavy Metal Transport and Detoxification

*Acidiella bohemica* encoded numerous genes related to heavy metal transport and detoxification to resist extremely environmental stress, such as a variety of heavy metal transporters, including copper transporter, magnesium transporter (ALR and MRS2), zinc transporter, arsenite efflux transporter (ArsB), and arsenite transporter (ACR3 family). They transported metal ions from the cytoplasm to the outside of cells. Some proteins related to detoxification also found in the genome, such as arsenical resistance protein (ArsH), arsenate reductase (Arc2), arsenite-translocating ATPases (ArsA), mercury reductase and cyanate lyase (cynS). Among them, the transcription activity of the gene encoding ArsA is relatively highest (FPKM value is 1,204) (Supplementary Table 5).

### Comparison of Genomes

**FIGURE 3:**
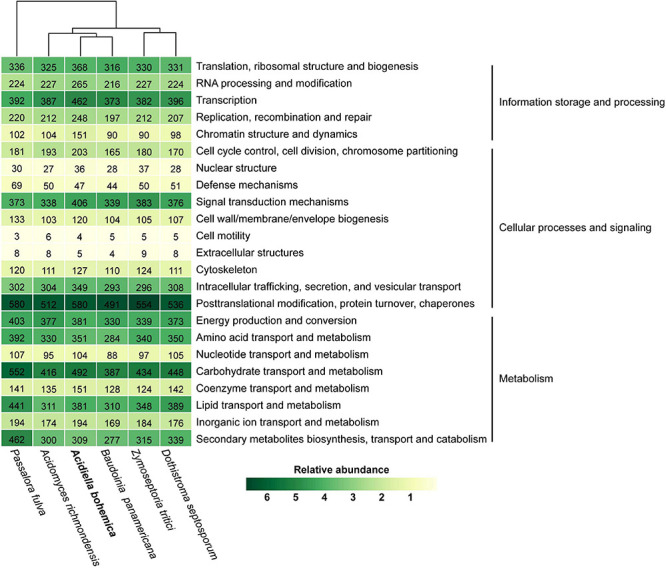
KOG classification of *A. bohemica* and five related fungi. Hierarchical clustering and heat map colors are based on percentage out of total number of proteins identified in the genome. The absolute number of proteins is given as labels.

As could be seen in Supplementary Figure 2, among the species with published genomes, *A. bohemica* and *B. panamericana* were closest to each other. The Teratosphaeriaceae family gathered into one branch, while the Mycosphaerellaceae family gathered into another. By comparison, the *A. bohemica* genome size and number of genes were larger than most species. In the *A. bohemica* genome, genes with different cell functions in large quantity were involved in post-translational translation, protein conversion, molecular chaperones and carbohydrate transport and metabolism. In gene composition, *A. bohemica* was closest to *B. panamericana* (Figure 3). Both of them had the same functional genes in the mechanisms of resistance to acid environment and heavy metal stress, indicating that their living niche might be the closest. In general, the abundance ratios of various types of genes in these species were not much different. Compared with other species, *A. bohemica* had the largest number of genes in information storage and processing. Moreover, *A. bohemica* also has more genes related to intracellular trafficking, secretion and vesicular transport, as well as inorganic ion transport, which may indicate that it has an important role in the absorption and transformation of nutrients in AMD environments. By comparative analysis of gene families, we found that four species harbored 5,966 homologous gene families that were mostly related to basic metabolism, indicating that these fungi were similar to *A. bohemica* (Supplementary Figure 3). Besides, *A. bohemica* had 162 unique gene families, most of which were predicted proteins encoding unknown functions. Some of these gene families had high transcriptional activities (FPKM reaches more than 100), and the eight families were annotated as encoding CAZymes.

**FIGURE 4:**
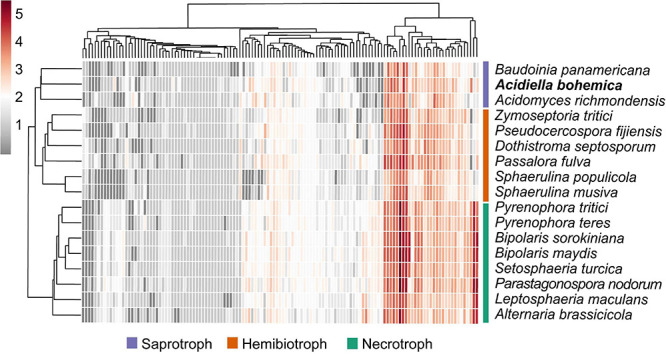
Heat map of CAZyme families in the *Dothideomycetes*. Both the CAZyme families and the species are hierarchically clustered. The clustering of organisms largely follows the phylogeny. The hemibiotrophs (orange) and saprotrophs (purple) within the *Capnodiales* cluster together, and the necrotrophs (green) within the *Hysteriales* and *Pleosporales* cluster together.

When we compared the *A. bohemica* genome with eight genomes of related species, the CAZyme family with the largest number of genes also exists widely in other species, such as GH3 and GH18. The *A. bohemica* genome does not contain a unique GH family, but GH7 and GH62 are slightly more than other species. *A. bohemica* showed more than the average number of genes encoding CAZyme among the compared genomes (Supplementary Figure 4). *A. bohemica*, *B. panamericana*, and *A. richmondens*is were more similar to other species in carbohydrates degradation (Figure 4). They differed from others belonging to *Capnodiales* in CBM1, AA7, GH109, GH62, GH6, GH27, and GH127. Obviously, *Capnodiales* and *Hysteriales* as well as *Pleosporales* are divided into two groups, based on CAZymes acting on degradation of cellulose, such as AA9. *Hysteriales* and *Pleosporales* had an average of 25 genes (maximum is 31, minimum is 20), while *Capnodiales* had only 1–3 genes. In addition to AA9, CBM1, AA7, CBM18, CBM50, GH45, GH7, and GH64 also showed the difference between the two groups. In addition to the differences in the main systematic classification of these taxa, the obvious difference was that *Capnodiales* had mostly hemibiotrophs (starting with live vegetative stage, and killing the plant host later) or biotrophs (forming a good connection with host plants and feeding on it). *A. bohemica*, *B. panamericana*, and *A. richmondensis* are all saprotrophs (decomposing dead organisms to obtain nutrients), while *Hysteriales* and *Pleosporales* are mostly necrotrophs (killing host plant cells and obtaining food sources). Therefore, we found that genes encoding carbohydrates degradation might be related to different lifestyles of fungi, such as the degradations of cellulose, xylan and pectin.

## Discussion

### Morphological Comparison and Biologically Induced Mineralization

In this study, *A. bohemica*, isolated from extreme AMD environments, is allied to *Dothideomycetes*, *Capnodiales*, and *Teratosphaeriaceae* in classification. It has been reported that *Dothideomycetes* was one of the largest fungal groups on the earth, with a high level of ecological diversity, including a large number of plant pathogens and a variety of infection host species (Ohm et al., 2012). The morphological analysis indicated that *A. bohemica* was a filamentous fungus, which was reproduced by conidia. The morphological characteristics of *A. bohemica* were basically consistent with those of the same species isolated from acid soil of coal mine (Hujslová et al., 2013). Phylogenetic and homologous analyses manifested that *A. bohemica* was closest related to *B. panamericana*. Both species belong to the same family (Teratosphaeriaceae). *B. panamericana* is a saprophytic fungus that can survive in extreme environments, inhabiting in various exposed substrates near cellar warehouses and commercial bakeries, where the ethanol vapor in the environment is conducive to its colonization (Scott et al., 2007). In addition to the great differences in habitat, both of their morphological characteristics were also diverse, which were mainly manifested in the shape of colonies and the color of hyphae (Scott et al., 2016). As far as we know, extreme acidophilic fungi grow optimally at pH <3, while moderately acidophilic fungi grow optimally at pH 3–5. The results of pH adaptability experiment confirmed that *A. bohemica* could grow optimally at pH 3, which could be classified as a moderate acidophilic fungus (Supplementary Figure 1). As another related species of *A. bohemica*, *A. richmondensis* is also an acidophilic fungus and isolated from AMD biofilms, optimally growing at pH 2–5. Different from *A. bohemica*, *A. richmondensis* has dark hyphae with a few branchings (Mosier et al., 2016). These proved that *A. bohemica* might be distinct from other species in morphology, although it was closely related to other species. However, morphological differences are of little significance as a basis for distinguishing *A. bohemica* and its related species in this study.

The results of simulated culture showed that the yellow precipitates formed during growth of *A. bohemica* contain high contents of iron, S, and K elements, which are the components of jarosite, a common mineral in AMD environments. Compared with non-inoculated control, the Fe elements in the culture solutions were greatly reduced by 20.7%, and the S and Ca elements were also remarkably reduced. Sulfide precipitation tends to remove more metals than hydroxide precipitation, because the solubility of sulfide is usually lower than that of hydroxide in a certain pH range. According to the XRD test results, we presumed that the precipitates were jarosite, copper hydroxide phosphate, gypsum, and anhydrite. Thus, we speculated that *A. bohemica* might have the potential of biologically induced mineralization. Biologically induced mineralization usually occurs when organisms change their local microenvironment through metabolic activities, or only due to the cell components such as cell walls, extracellular polymers or even spores, which produce charged surfaces as adsorption sites for ions from the environments, resulting in the nucleation and growth of minerals (Oggerin et al., 2013). In this study, *A. bohemica* seems to grow very slowly. Only some needle like substances are on the cell surface after a month; while in the study of Oggerin et al. (2013), the fungal cell surface can be wrapped with thick minerals. Moreover, our XRD test results failed to have a good result, which may be due to the excessive organisms in the precipitate, causing the poor crystalline state of the minerals.

### Metabolisms of Carbon, Nitrogen, and Sulfur

The results of gene prediction and functional annotation we obtained confirmed that there are many genes encoding CAZymes in the genome. Enzymes involved in carbohydrate decomposition show remarkably functional diversity. A great many of genes participating in the hydrolysis fructose, mannose, galactose, starch, and sucrose were found in the *A. bohemica* genome (Supplementary Table 5). These genes play a key role in the hydrolysis of sugars in oligotrophic AMD environments, which is common in AMD microorganisms. For instance, the first-documented acidophilic archaeon, *Sulfolobus acidocaldarius* (Brock et al., 1972), *Sulfolobus solfataricus* (Zillig et al., 1980), and the related species, *A. richmondensis* (Mosier et al., 2016) were confirmed that they can grow on complex organic substrates such as mono-, dis-, and polysaccharides. Furthermore, *A. bohemica* genome encoded the EMP pathway, the pentose phosphate pathway, the TCA and the glyoxylate bypass in aerobic respiration for degradation of organic acids to obtain energy. In fact, most bacteria and archaea in AMD environments can use the reductive TCA cycle for carbon assimilation, such as the phylum Aquificae (Hügler et al., 2007) and a new deltaproteobacterial order Candidatus Acidulodesulfobacterales (Tan et al., 2019). However, few acidophilic fungi (belong to the order Capnodiales) can perform the reductive TCA cycle. The mainly probable reason is that they have different nutritional patterns. The former is obligate or facultative autotroph, while the latter (belong to the order Capnodiales) is heterotrophic (Barrie and Hallberg, 2009). In general, the *A. bohemica* can decompose macromolecular carbon to obtain energy for life activities.

Nitrate is an important inorganic nitrogen source available to microorganisms. *A. bohemica* can participate in the nitrate assimilation, but nitrogen fixation cannot be carried out. In *A. bohemica*, glutamate can form glutamine under the catalysis of glutamine synthetase (glnA) with NH_4_^+^ produced during the assimilation of nitrate. Glutamine will become the source of amino acids in biosynthesis, ultimately it will be involved in the synthesis of nucleic acids and proteins in microorganisms. *A. bohemica* has a P450nor protein that is one of the key enzymes in denitrification pathway of fungi for catalyzing NO to form N_2_O. But no nitrite reductase, such a NirK (catalyzing the formation of nitrite to NO), in denitrification pathway has been found. However, the genes encoding NirK and P450nor are expressed in *A. richmondensis* (Mosier et al., 2016). In the past, many fungi have been confirmed to have obvious denitrification activity (Shoun et al., 1992). The most significant feature of fungal denitrification system is P450nor involved in the reduction of NO to N_2_O. Fungi can utilize the nitrite produced by nitrate reduction in the assimilation pathway for denitrification, which generally does not generate N_2_ (Shoun et al., 2012). Therefore, we conjectured that *A. bohemica* could potentially conduct the nitrate assimilation and denitrification.

Sulfate is the main form of sulfur in nature, readily assimilated by organisms. SO_4_^2–^ is reduced to sulfide and ultimately integrated into cysteine in the pathway of sulfate assimilation. *A. bohemica* has related genes encoding the enzymes involved in assimilation sulfate reduction (Supplementary Table 5). These genes are also found in the transcriptomic data. In *A. bohemica*, two sulfate-activating enzymes, sulfate adenylyltransferase (sat) and adenylylsulfate kinase (cysC), catalyze the activation of sulfate to adenosine-5′-phosphosulfate (APS) and then to 3′-phosphoadenosine 5′-phosphosulfate (PAPS). Phosphoadenosine phosphosulfate reductase (cysH) and sulfate reductase (NADPH) flavoprotein and hemoprotein (cysJ and cysI) engaged in the reduction of PASA to sulfite and then to S^2–^. Finally, S^2–^ is incorporate into cysteine, which is encoded by cysteine synthase A (cysK) (Leyh, 1993; Marzluf, 1997). Cysteine plays a critical role in the synthesis of methionine, whereas both of them are essential for the growth and activities of all cells. Thus, sulfate assimilation is significant for the synthesis of cysteine and methionine. In addition, no genes related to dissimilatory sulfate reduction and sulfur oxidation were found in *A. bohemica*. Dissimilatory sulfate reduction usually occurs in obligate or facultative anaerobes (Barrie and Hallberg, 2009), such as *Ca*. Acidulodesulfobacterales (Tan et al., 2019). Therefore, *A. bohemica* is not an anaerobic fungus and does not have the ability to oxidize sulfur.

### Iron Metabolism

**FIGURE 5:**
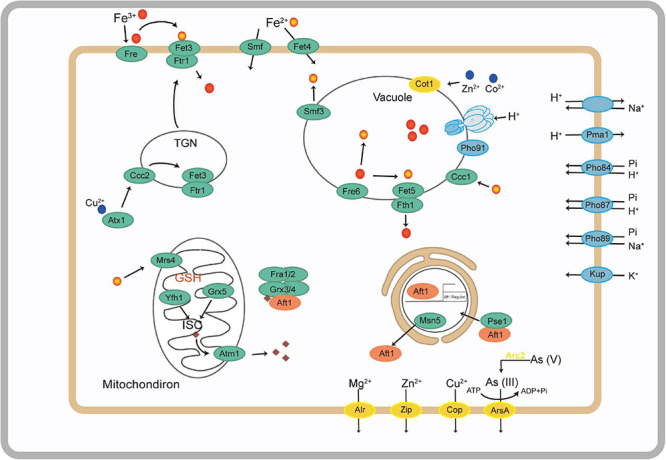
Metabolic abilities associated with putative iron utilization pathway (green), stress response (blue), and other metal transporters (yellow) of *A. bohemica* based on the genes predicted from its draft genome.

Iron has great significance in the growth and reproduction of microorganisms, normally existing in the form of heme and iron-sulfur clusters. However, the distribution of iron in the environment is not uniform. There may be competition with other microorganisms, which leads to the disproportionate dissolution and absorption of iron (Robinson et al., 2021). Generally, there are two methods of iron uptake: reductive and non-reductive uptake systems on the surface of fungal cells. Non-reductive uptake system is dependent on siderophore-iron chelates, which is widespread in most of fungi, for example, the two subclasses of *Ascomycota* including *Neurospora crassa* (Euascomycetes) and *Candida albicans* (Hemiascomycetes). They can synthesize and secrete siderophore to specifically binding to ferric iron (Kosman, 2003). Nevertheless, there is no gene related to non-reductive uptake system that has been found in the *A. bohemica* genome. The *A. bohemica* genome encoded genes related to the reductive systems of iron uptake (Figure 5). Ferric iron in the external environment is first reduced to absorbable ferrous iron by the Fre on the plasma membrane. Before being absorbed, ferrous iron needs to be oxidized, which requires oxidizing activity of Fet3. Then ferric iron is transferred to the cytoplasm through permease Ftr1. After transported into the cells, excess Fe^3+^ are stored in the vacuoles, which may interact with amino and polyphosphoric acids. Ccc1 on the vacuolar membrane transports iron and manganese exclusively. The transporters associated with iron on the vacuole membrane are similar to the plasma membrane system. Before transported out of vacuoles, the iron needs to be reduced by Fre6. In general, Fet5/Fth1 and Smf3 can transport the ferrous iron reduced by Fre6, impelling iron from vacuole into cytoplasm. In this process, the presence of Fre6 also indicates that the iron in vacuoles is in the form of ferrous. Vacuoles are the places where many metal ions are stored, including iron, zinc, copper, and manganese. Large amounts of iron may also be stored through Mrs4 in the mitochondria that require Yfh1, Grx5, and glutathione to synthesize iron-sulfur clusters. Yfh1 can be conducive to form polymers of up to 60 subunits, consuming more than 3,000 iron atoms (Kaplan et al., 2006; Philpott, 2006; Ramos-Alonso et al., 2020). The *A. bohemica* genome also encoded Aft, which is regarded as the regulator of iron balance.

### Mechanisms of Response to Extreme Environment

#### Response to Acid Stress

Acid mine drainage is an extreme acid environment and low pH will threaten the growth of microorganisms (Rothschild and Mancinelli, 2001; Huang et al., 2016; Distaso et al., 2020). In order to adapt to acid stress, microorganisms will form a series of defense mechanisms to regulate intracellular pH, K^+^, and Na^+^ concentration. *A. bohemica* maintains intracellular pH homeostasis in the following ways: (1) positive potential in the cell membrane caused by K^+^ influx; (2) transport extra protons outside the cell through ATPase, antiporter, and cotransporter (Supplementary Table 5); (3) cytoplasmic buffering (such as glutamic and phosphoric acids); and (4) degradation of organic acids. Generally, the potential inside the cell membrane is negative at resting state of cells, and the potential outside the membrane is positive. Eosinophils may be able to prevent the inflow of protons by the opposite membrane potential, which is mainly generated by the influx of potassium ions (Tyson et al., 2004; Baker-Austin and Dopson, 2007). The genomic and transcriptomic data confirmed that *A. bohemica* has the proton efflux systems to pump excess protons out of the cells for maintaining pH balance. The buffering effect of the cytoplasm is capable of maintaining the intracellular pH. Firstly, the buffering effect of the cytoplasm can remove or release H^+^. For example, glutamate in cells conducts decarboxylation under the glutamate decarboxylase to release H^+^. The other buffer is H_3_PO_4_ (pKa = 7.2). The *A. bohemica* genome encoded Pho84, Pho87, Pho89, and Pho91 on the vacuole membrane, which are essential for the storage and usage of polyphosphates (Chen et al., 2015). Organic acids (such as acetic and lactic acids) are harmful to cells because they inhibited the respiratory chain at low pH (Ciaramella et al., 2005). The *A. bohemica* genome encoded propionyl-CoA synthase and acetyl-CoA synthetases to degrade organic acids for pH stability (Baker-Austin and Dopson, 2007). The *A. bohemica* genome contains genes related to DNA and protein repair. The existence of these genes may be related to the maintenance of intracellular pH balance. Chaperones can repair DNA and protein damage caused by low pH.

#### Transport and Detoxification of Heavy Metals

Ordinarily, there are two mechanisms of fungi tolerance to heavy metals: extracellular and intracellular sequestration. Extracellular mechanisms prevent metals entry by biosorption or releasing organic molecule to chelate metal ions. In the intracellular mechanism, heavy metals are combined with metal transport proteins or other ligands to extrude metal ions from the cytosol out of the cell or by transporting metal ions into vacuolar (Anahid et al., 2011; Chan et al., 2018; Glukhova et al., 2018). In *A. bohemica*, the second mechanism is found to adapt to the environment of high heavy metal concentration. *A. bohemica* genome encoded proteins related to some heavy metals resistance mechanisms to resist extreme environmental stresses, such as a variety of heavy metal transporters (Supplementary Table 5). They transport metal ions from the cytoplasm to the outside of the cell. Some proteins related to detoxification reactions were also found in the genome. The presence of these proteins infers that *A. bohemica* may have adaptability in AMD environments with high content of heavy metals.

## Conclusion

In this study, we isolated and cultured *A. bohemica* from an AMD environment. The whole genome of *A. bohemica* was sequenced by PacBio single-molecule real-time sequencing. It was the first time to release on the genomic traits of this acidophilic fungus. Our study indicated that *A. bohemica* might have the ability of biologically induced mineralization. Genomic traits in response to low pH and heavy metals confirm its unique tolerance mechanism enabling the *A. bohemica* to survive across extremely environmental stresses. Meanwhile, we reconstructed the C, N, S, and Fe metabolic pathways to reveal the life process of *A. bohemica* based on genomic and transcriptomic data. These findings broaden our understanding of the acidophilic fungi, *A. bohemica* in AMD environments from the aspects of morphology, physiology and genome. In the future, we look forward to such a study route can be helpful for the identification of microorganisms in AMD environments.

## Data Availability Statement

The datasets presented in this study can be found in online repositories. The names of the repository/repositories and accession number(s) can be found below: https://www.ncbi.nlm.nih.gov/, PRJNA725650.

## Author Contributions

PJ, S-NO, J-LL, J-TL, and W-SS developed and framed the research questions. S-NO, J-LL, X-MJ, and BL conducted the experiments and collected the data. S-NO, J-LL, and PJ performed the data analyses. S-NO, J-LL, and PJ wrote the first draft of the manuscript. All authors revised the manuscript.

## Conflict of Interest

The authors declare that the research was conducted in the absence of any commercial or financial relationships that could be construed as a potential conflict of interest.

## Footnotes

1 http://www.cazy.org
