# Supplementary material for: Physiological, Genomic and Transcriptomic Analyses Reveal the Adaptation Mechanisms of Acidiella bohemica to Extreme Acid Mine Drainage Environments
Source: Front Microbiol. 2021 Jul 8;12:705839. doi: 10.3389/fmicb.2021.705839 (PMC8298002; doi:10.3389/fmicb.2021.705839)
Supplement: Supplementary file 2 [file Data_Sheet_1.docx]

Supplementary Material

**Supplementary Table 1**. List of studied acidophilic fungi in extremely acidic environments.

| Species | Source | pH | Geography | Genome available | Reference |
| --- | --- | --- | --- | --- | --- |
| *Scytalidium acidophilium* | Solution containing 4% copper sulfate | 0.2–0.7 | Danmark | Yes | Starkey and Waksman, 1943 |
| *Acontium velatum* | Solution containing 4% copper sulfate | 0.2–0.7 | Danmark | No | Starkey and Waksman, 1943 |
| *Penicillium corylophilum* | Acid mine water | 2 | USA | No | Sinclair and Herring 1975 |
| *Aspergillus* sp. P37 | Rio Tinto river with high acidity and heavy metal concentrations | 2–2.3 | Spain | No | Cánovas et al., 2003 |
| *Acidomyces richmondensis* | Acid mine drainage | 0.8–1.38 | USA | Yes | Baker et al., 2004 |
| *Hortaea acidophila* | Brown coal containing humic and fulvic acids | 0.6 | Germany | No | Holker et al., 2004 |
| *Acidomyces acidophilus* | Soil adjacent to a sulphur pilefield from a natural gas purification plant | 1.4–3.5 | Canada | No | Selbmann et al., 2008 |
| *Bispora* sp. MEY-1 | Acidic waste water of the 721 uranium mine | 2.5–3.0 | China | Yes | Luo et al., 2009 |
| *Teratosphaeria acidotherma* | Microbial mats and biofilms from extremely acidic and high temperature hot spring. | 1.5 | Japan | No | Yamazaki et al., 2010 |

**Supplementary Table 1**. List of studied acidophilic fungi in extremely acidic environments (continued).

| Species | Source | pH | Geography | Genome available | Reference |
| --- | --- | --- | --- | --- | --- |
| *Acidiella bohemica* | Extremely acidic soil | 2.2–2.5 | Czech Republic | No | Hujslová et al., 2013 |
| *Acidea extrema* | Highly acidic soil | 2 | Czech Republic | No | Hujslová et al., 2014 |
| *Acidothrix acidophila* | Highly acidic soil | 1.8 | Czech Republic | No | Hujslová et al., 2014 |
| *Soosiella minima* | Highly acidic soil | 2 | Czech Republic | No | Hujslová et al., 2014 |
| *Penicillium* spp. | A metal mine | 2.5 | Russia | No | Glukhova et al., 2018 |

**Supplementary Table 2.** Accession numbers (NCBI) of 13 compared species.

| Species | Accession number |
| --- | --- |
| *Acidiella bohemica* SYSU C17045 | PRJNA725650 |
| *Acidomyces richmondensis* BFW | PRJNA207869 |
| *Aspergillus niger* ATCC 1015 | PRJNA15785 |
| *Aureobasidium pullulans* EXF-150 | PRJNA207874 |
| *Aureobasidium namibiae* CBS 147.97 | PRJNA207872 |
| *Aureobasidium subglaciale* EXF-2481 | PRJNA161477 |
| *Baudoinia panamericana* UAMH 10762 | PRJNA53579 |
| *Dothistoma septosporum* NZE10 | PRJNA74753 |
| *Passalora fulva* CBS 131901 | PRJNA86753 |
| *Pseudocercospora fijiensis* CIRAD86 | PRJNA19049 |
| *Sphaerulina populicola* P02.02b | PRJNA81737 |
| *Sphaerulina musiva* SO2202 | PRJNA51781 |
| *Zymoseptoria tritici* IPO323 | PRJNA19047 |

**Supplementary Table 3.** Content of partial element of the culture media (mg/L).

|  | Fe (II) | Total Fe | S | Ca |
| --- | --- | --- | --- | --- |
| Inoculated culture | 151 ± 28.5 | 893 ± 50.1 | 1336 ± 46.0 | 498 ± 71.7 |
| Non-inoculated control | 281 ± 20.9 | 1127 ± 36.4 | 1565 ± 104 | 605 ± 47.0 |

**Supplementary Table 4.** General features of the *A. bohemica* genome sequence.

|  | Primary contig | Haplotig |
| --- | --- | --- |
| Contigs | 62 | 181 |
| Total length (bp) | 26,833,561 | 23,156,087 |
| GC (%) | 57 | 57 |
| N50 (bp) | 1,164,001 | 498,020 |
| CEGMA | 96.4% | 84.7% |
| BUSCO (ascomycetes) | 95.8% | 86.2% |


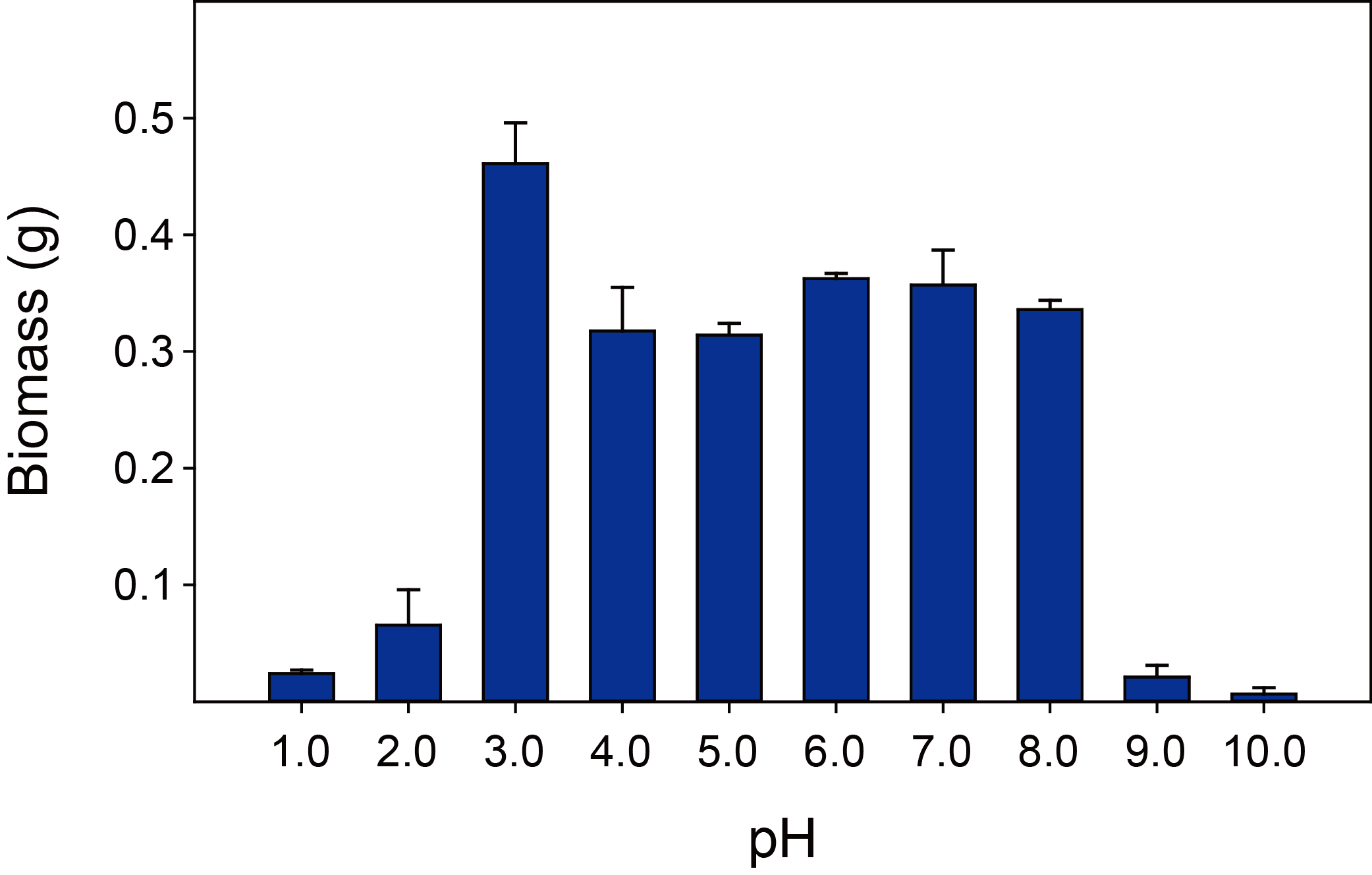


**Supplementary Figure 1.** Growth of *A. bohemica* in PDA liquid medium at different pH values after 7 days of incubation at 25 ℃.


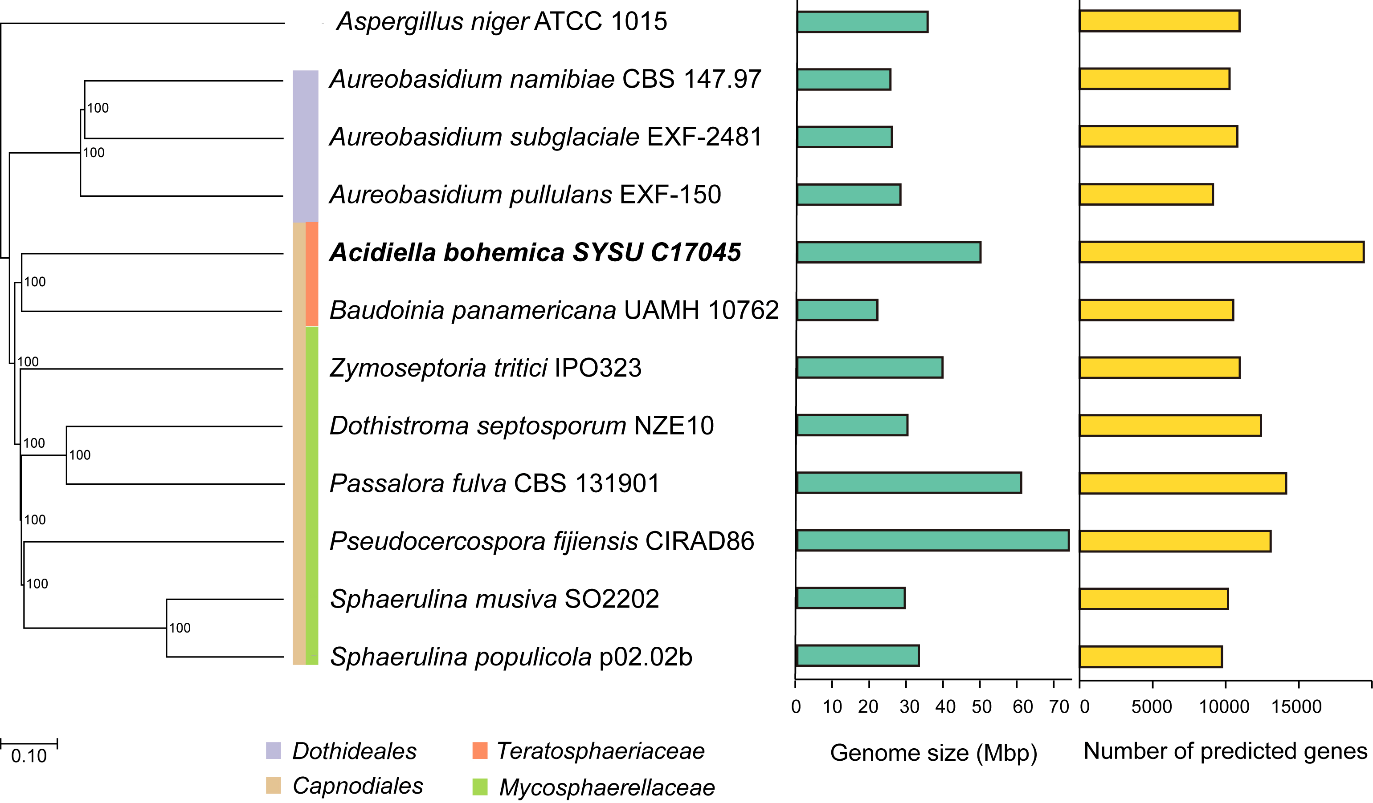


**Supplementary Figure 2.** The proteome FFP tree at feature length l = 13 and genome characteristics. The tree was constructed with neighbor joining. All clades have 100% jackknife monophyly index, which means that the clade always exists in jackknife FFP trees. *Aspergillus niger* was used as an outgroup and its branch on the tree is not drawn to scale.


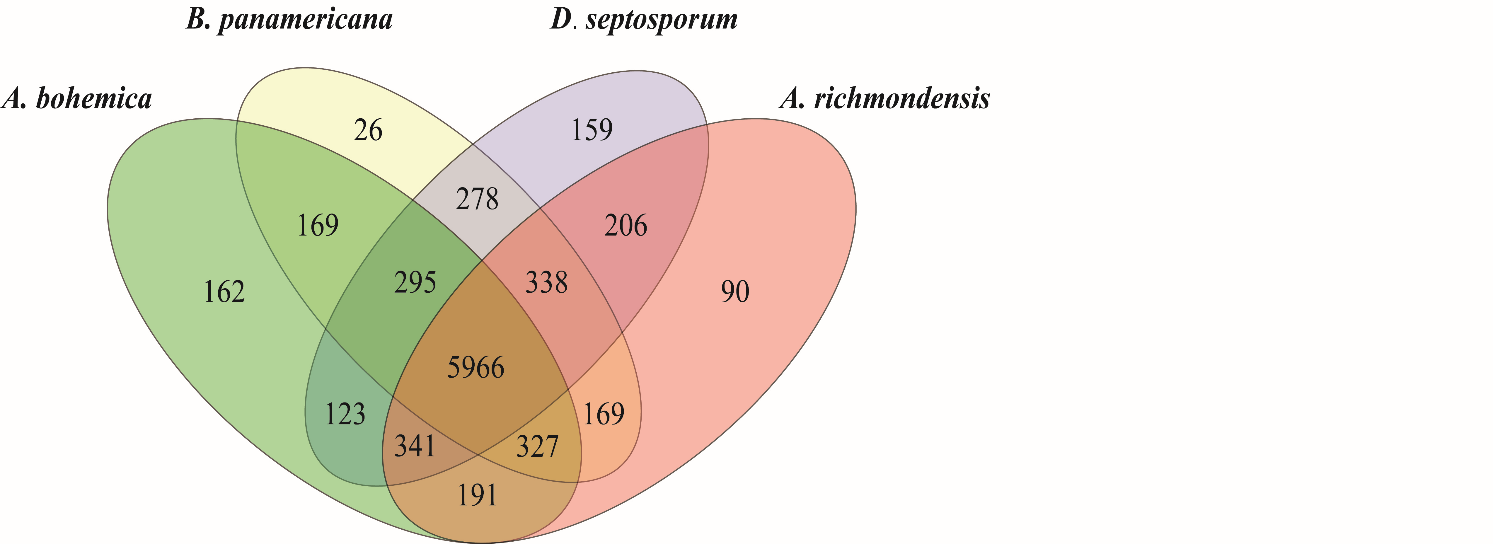


**Supplementary Figure 3.** Venn diagram showing unique and shared orthologous gene families between and among the four closely related *Dothideomycetes* fungi. The orthologous gene families among *Acidiella bohemica*, *Baudoinia panamericana*, *Dothistroma septosporum* and *Acidomyces richmondensis* were identified using OrthoMCL. 5,966 gene families are orthologous in all the four fungi.


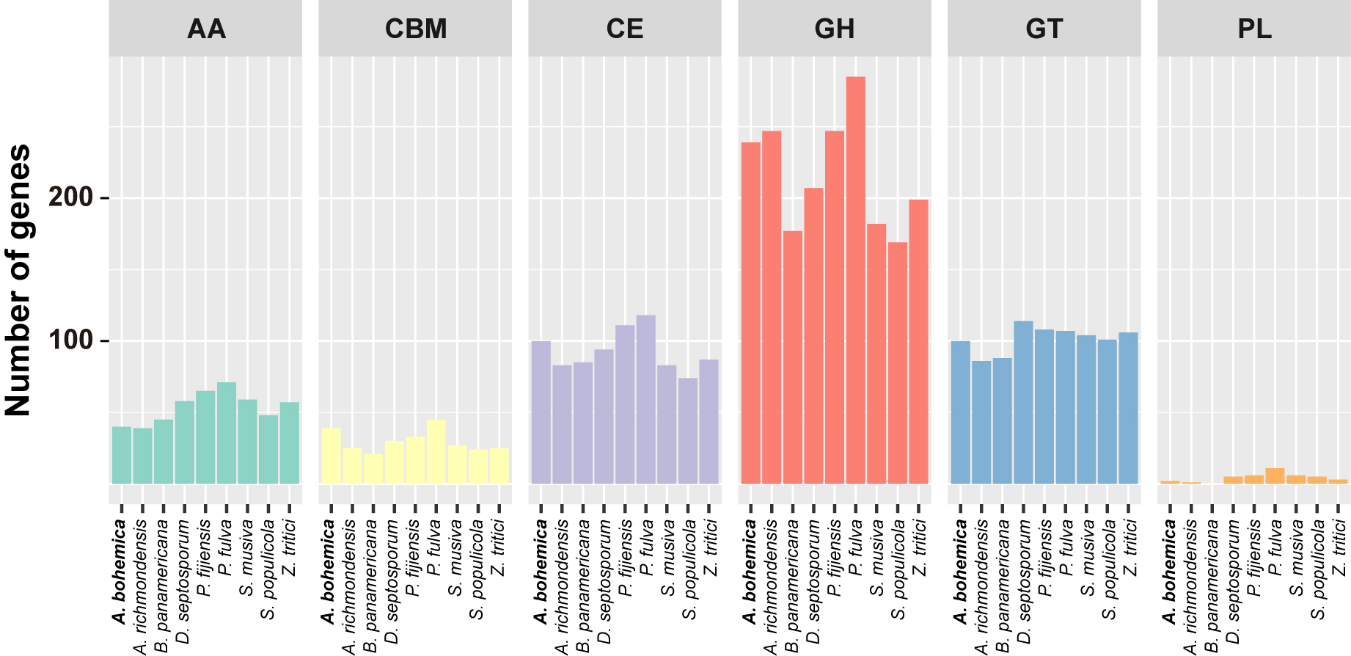


**Supplementary Figure 4.** Annotation of CAZymes in *A. bohemica* and 8 related fungi. CAZymes are grouped into the classes: glycoside hydrolases (GH), glycosyl transferase (GT), carbohydrate esterases (CE), polysaccharide lyases (PL) and auxiliary activities (AA) and carbohydrate-binding modules (CBM).
