# Supplementary figures and images for: Physiological, Genomic and Transcriptomic Analyses Reveal the Adaptation Mechanisms of Acidiella bohemica to Extreme Acid Mine Drainage Environments
Source: Front Microbiol. 2021 Jul 8;12:705839. doi: 10.3389/fmicb.2021.705839 (PMC8298002; doi:10.3389/fmicb.2021.705839)

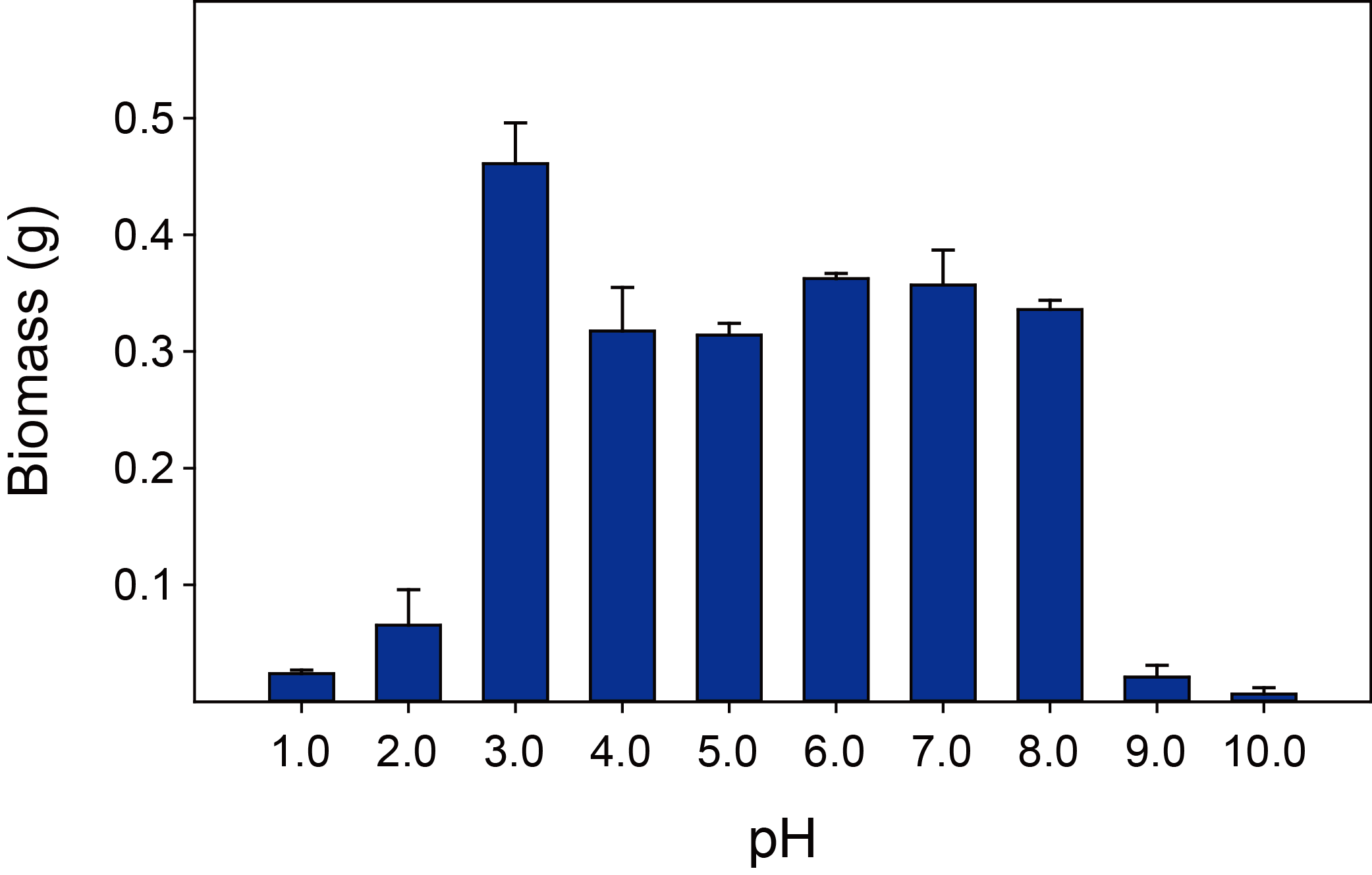

Supplement: Supplementary file 3 [file Image_1.TIF]

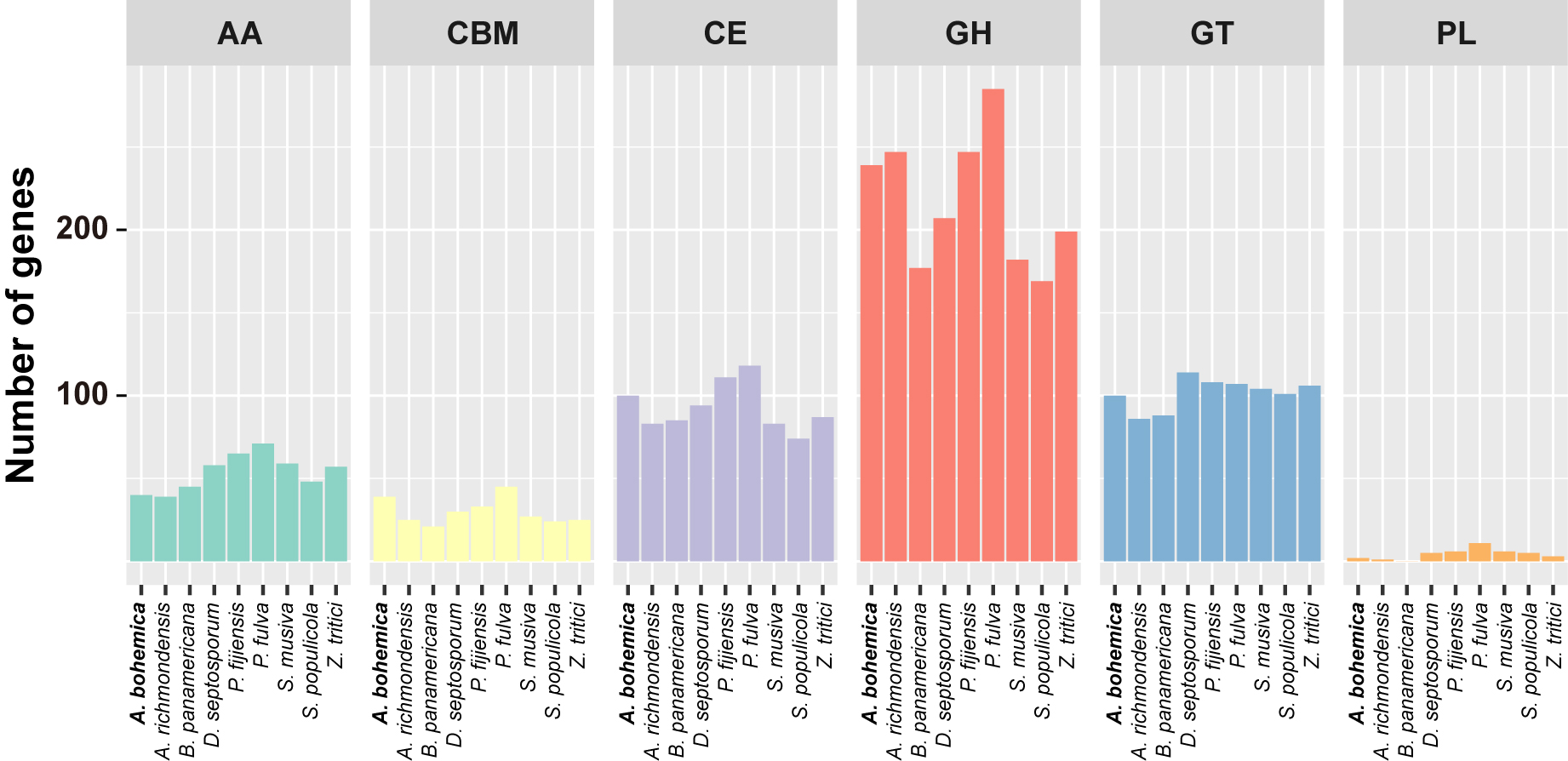

Supplement: Supplementary file 4 [file Image_2.TIFF]

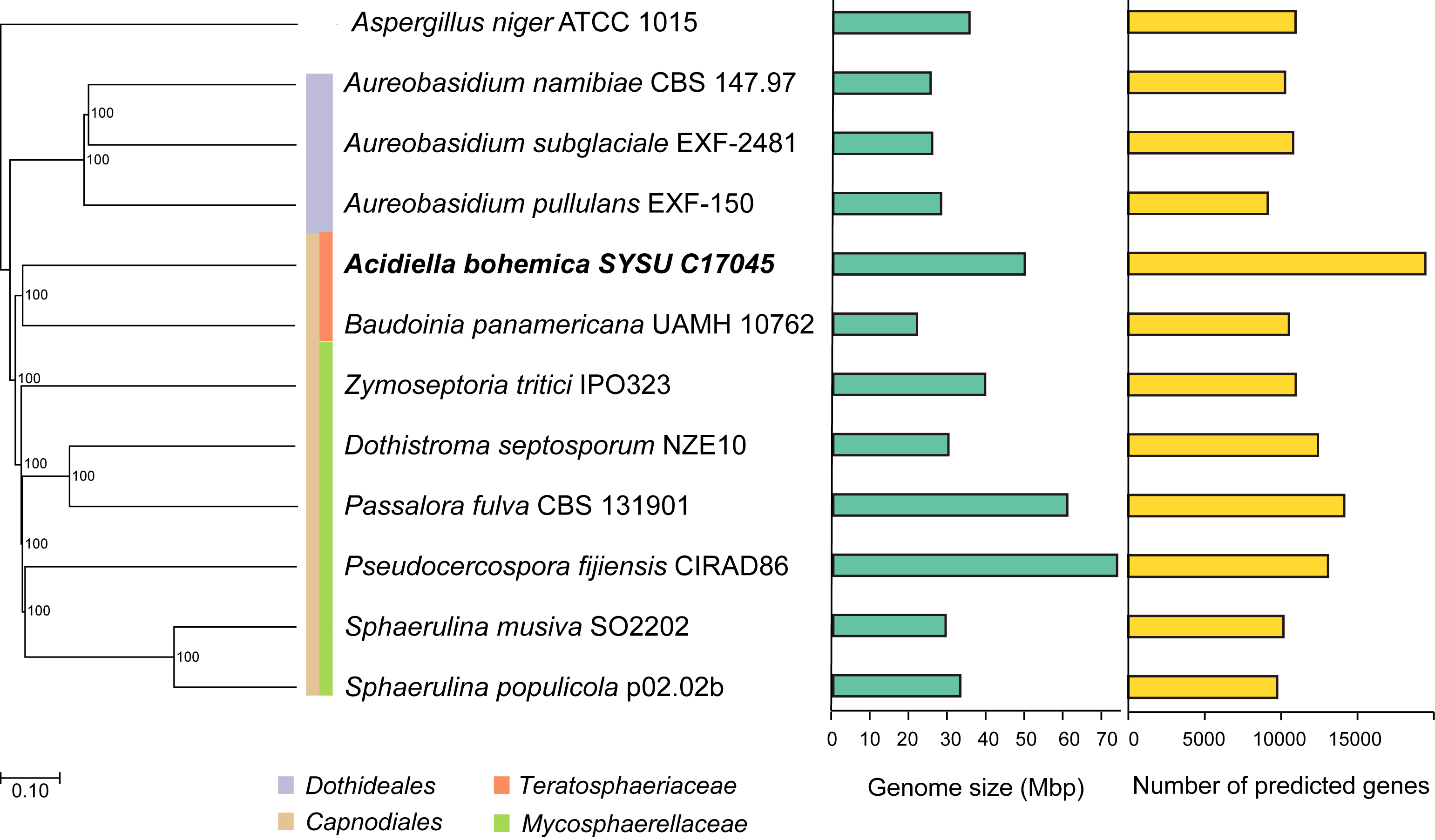

Supplement: Supplementary file 5 [file Image_3.TIFF]

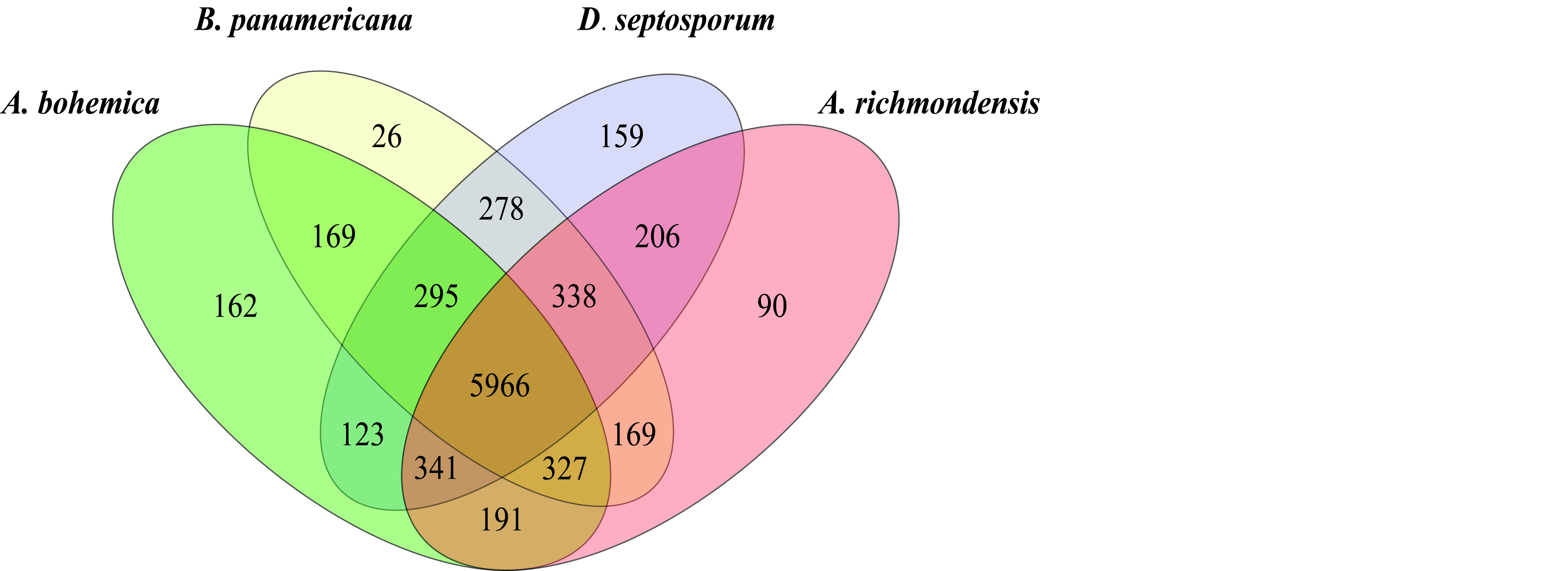

Supplement: Supplementary file 6 [file Image_4.TIF]
